# Capturing the Trajectory of Psychological Status and Analyzing Online Public Reactions During the Coronavirus Disease 2019 Pandemic Through Weibo Posts in China

**DOI:** 10.3389/fpsyg.2021.744691

**Published:** 2021-09-29

**Authors:** Yi-Chen Chiang, Meijie Chu, Shengnan Lin, Xinlan Cai, Qing Chen, Hongshuai Wang, An Li, Jia Rui, Xiaoke Zhang, Fang Xie, Chun-Yang Lee, Tianmu Chen

**Affiliations:** ^1^State Key Laboratory of Molecular Vaccinology and Molecular Diagnostics, School of Public Health, Xiamen University, Xiamen, China; ^2^Nanjing Terlton Information Technology Co. Ltd., Nanjing, China; ^3^Beijing Hongbo Zhiwei Technology Co. Ltd., Beijing, China; ^4^School of International Business, Xiamen University Tan Kah Kee College, Zhangzhou, China

**Keywords:** COVID-19, public opinion, psychological status, social media, public reactions, China

## Abstract

When a major, sudden infectious disease occurs, people tend to react emotionally and display reactions such as tension, anxiety, fear, depression, and somatization symptoms. Social media played a substantial awareness role in developing countries during the outbreak of coronavirus disease 2019 (COVID-19). This study aimed to analyze public opinion regarding COVID-19 and to explore the trajectory of psychological status and online public reactions to the COVID-19 pandemic by examining online content from Weibo in China. This study consisted of three steps: first, Weibo posts created during the pandemic were collected and preprocessed on a large scale; second, public sentiment orientation was classified as “optimistic/pessimistic/neutral” orientation *via* natural language processing and manual determination procedures; and third, qualitative and quantitative analyses were conducted to reveal the trajectory of public psychological status and online public reactions during the COVID-19 pandemic. Public psychological status differed in different periods of the pandemic (from December 2019 to May 2020). The newly confirmed cases had an almost 1-month lagged effect on public psychological status. Among the 15 events with high impact indexes or related to government decisions, there were 10 optimism orientation > pessimism orientation (OP) events (2/3) and 5 pessimism orientation > optimism orientation (PO) events (1/3). Among the top two OP events, the high-frequency words were “race against time” and “support,” while in the top two PO events, the high-frequency words were “irrationally purchase” and “pass away.” We proposed a hypothesis that people developed negative self-perception when they received PO events, but their cognition was developed by how these external stimuli were processed and evaluated. These results offer implications for public health policymakers on understanding public psychological status from social media. This study demonstrates the benefits of promoting psychological healthcare and hygiene activity in the early period and improving risk perception for the public based on public opinion and the coping abilities of people. Health managers should focus on disseminating socially oriented strategies to improve the policy literacy of Internet users, thereby facilitating the disease prevention work for the COVID-19 pandemic and other major public events.

## Introduction

Mental health disorders impose an enormous disease burden on societies worldwide. Depression alone affects 350 million people globally and is the leading cause of disability worldwide (The World Bank, 2020). When a major, sudden infectious disease or social unrest outbreak occurs, people are prone to react emotionally and display behavioral reactions such as tension, anxiety, fear, depression, and somatization symptoms due to the suddenness, danger, uncertainty, and environmental changes associated with the event (Ni et al., 2020; Shigemura et al., 2020). Especially when the cause of the pandemic is unknown, rumors are often prevalent (Ren et al., 2020), and the uncertainty and the asymmetry of the information flow can easily cause people to speculate, panic, worry, or take unsupported preventive measures. Some people may even engage in consistent blind obedience. To develop appropriate strategies for improving public rational reactions and coping patterns, it may be important to understand the effect of a major emergency on the emotional reactions of people.

The epidemic of the severe acute respiratory syndrome (SARS) in 2003 exerted a substantial impact on society. At the beginning of the outbreak in China, community tension increased, and rumors regarding the pandemic spread through oral communication, mobile phone text messages, and social media, which intensified the spread of social panic (Qiu et al., 2018). The incidence of acute posttraumatic stress disorder (PTSD) in patients who had recovered from SARS was 9.79% (Yan et al., 2004). After the outbreak of the Middle East respiratory syndrome (MERS) in South Korea, a total of 63.5% of the survivors suffered from severe mental illness, including posttraumatic symptoms (36.5%), sleep problems (36.5%), anxiety disorders (30.9%), and depression (30.2%) (Shin et al., 2019). The coronavirus disease 2019 (COVID-19) pandemic has now become a global public health crisis. This study focused on public psychological status and public reactions during the COVID-19 pandemic.

During the COVID-19 pandemic, public concern increased regarding the effects of the novel coronavirus and its impact on their economic and social situation and physical and mental health (Bol, 2021). A questionnaire survey indicated that the outbreak of COVID-19 could slightly affect the emotional reactions of public (Li J. B. et al., 2020). Mental stress has become a serious global public health issue accentuated by the COVID-19 pandemic (Javed et al., 2020; Pfefferbaum and North, 2020). The mental health symptoms in China during the COVID-19 pandemic have been prevalent (Zheng et al., 2021). During the pandemic, people have been experiencing psychiatric symptoms and disorders, such as fear, stress, anxiety, disappointment, irritability, depressive disorders, and even suicide (Dong and Bouey, 2020; Goyal et al., 2020; Ho et al., 2020; Hossain et al., 2020; Li W. et al., 2020; Dube et al., 2021). A meta-analysis found a high prevalence of acute stress and fear symptoms in the early period of the pandemic, while people may feel anxiety and depression symptoms during the more recent stages of the pandemic (Li et al., 2021).

Social media provided new channels for news reporting and created opportunities for the public to express their opinions and attitudes instantly through the comments of the readers (Bergstrom and Wadbring, 2015). Since the implementation of quarantine and social distancing measures, people have been increasingly receiving news and expressing opinions on social media platforms. Social media played a substantial awareness role during the outbreak of COVID-19 (Abdul-Baki et al., 2020). The media is important in formulating public health-protective behavior (Keren et al., 2021). Authentic, effective information communication can reduce public tension, anxiety, panic, and suspicion and can curb the spread of rumors. Negative emotion is associated with risk perception and coping responses (Gan et al., 2021). Previous studies have conducted a sentiment analysis to explore the responses and perceptions of individuals regarding the public on the COVID-19 outbreak through the examination of media platforms, such as Twitter, Facebook, Instagram, Weibo, and YouTube (Li Y. et al., 2020; Shorey et al., 2020; Tri Sakti et al., 2021). People may have more negative emotions related to the overall outbreak, but the public sentiments tend to be positive regarding some public health measures, the level of transparency of the Chinese government, and the outbreak response of the government (Xu et al., 2020; Jang et al., 2021).

This study was based on the development over time of the pandemic and two important strategies implemented by the Chinese government (i.e., the Wuhan lockdown on January 22nd and the lifting of lockdown on April 7). According to the pre-crisis, crisis, and postcrisis theory put forward in the life cycle of crisis suggested by Guth (Guth, 1995), the COVID-19 pandemic prevention and control work in China can be divided into three periods: the period of information uncertainty (from December 30, 2019, to January 22, 2020), the outbreak attention period (from January 23, 2020, to April 7, 2020), and the regular epidemic prevention and control period (after April 8, 2020). A previous study analyzed online public sentiment during the early stages of the COVID-19 pandemic (Xu et al., 2020). Some studies have analyzed online public emotions during the COVID-19 pandemic in several Chinese provinces or cities (Li Y. et al., 2020; Su et al., 2020). This study revealed the online public psychological status in China during the first wave of the pandemic, including the period of information uncertainty, the outbreak attention period, and the regular epidemic prevention and control period, and investigates the simultaneous and lagged effects of confirmed public emotions of COVID-19 cases.

After the initial occurrence of COVID-19, countries implemented strict prevention and control measures to curb the spread of the disease (Adhikari et al., 2020). China rapidly adopted a series of strategies to prevent and control the development of the pandemic, utilized effective information from public opinion monitoring, and appeased public sentiment. Tan Desai, the director general of the WHO, highly praised the response strategies of China (World Health Organization, 2020b), and many countries spoke highly of the pandemic prevention and control work of China and its contribution to global public health. Government measures to control the COVID-19 pandemic, effective communication, and public reactions are vitally important for coping with the pandemic and reducing the death rate (Thewall and Thelwall, 2020; Bol, 2021). If the public understands the situation of the COVID-19 pandemic and complies with certain restrictive measures, everyone in the country can be involved in dealing with the crisis process. Therefore, it is important to explore public emotional reactions to these anti-epidemic strategies during the pandemic. A previous study analyzed the emotions of Internet users on social distancing, lockdown, home confinement, and quarantine (Su et al., 2020; Ma et al., 2021; Shen et al., 2021; Wu et al., 2021). However, studies that explore the difference of public emotions expressed by social media users toward a series of anti-epidemic strategies and major events, which received more attention and discussion during the pandemic at the national level in China, are limited.

Previous studies used the data from Twitter to analyze the general public reactions to the COVID-19 pandemic (Zou et al., 2020; Noor et al., 2021). Some scholars have used a machine learning approach to illustrate the topics that were most frequently discussed by the public during the pandemic. People may pay attention to public health measures, social stigma, newly confirmed COVID-19 cases and deaths, the global pandemic situation, and gratitude to government and health workers (Xue et al., 2020; Zou et al., 2020). If public opinion is out of control, social governance, and national development will be seriously affected. It is therefore critical to promote public opinion in a favorable direction benefiting pandemics for prevention and control. To help public health agencies communicate health policies or messages and promote public mental health during the crisis, the patterns of public reactions during the pandemic require more attention.

The anxiety theory suggested by Edith Jacobson consists of four factors, namely, stimulus, self-perception, ability, and cognition (Tuttman, 1985). The stimulus may be an internal, instinctive impulse or external environment. Self-perception is defined as views of individuals themselves or any of their mental or physical attributes (Pam, 2013). The self-perception theory indicates that the emotional feelings of people are based on the situations in which they find themselves or on their own actions (Laird and Bresler, 1992). People can develop inner feelings of continuity of the self and can develop the ability to distinguish and compare the features of different objects. Cognition in the fields of social psychology is used to explain attitudes, attribution, and group dynamics (Sternberg and Sternberg, 2009). The essence of anxiety is that individuals cannot choose the behaviors that they prefer to release tension. The individuals feel the threat from internal and external stimuli to themselves through cognitive evaluation, and they may feel anxiety when they are unable to cope with the situation (Cai, 1995).

Based on the communication model suggested by Lasswell, the whole communication process consists of five elements: the sender, the message, the channel used, the audience, and the effect (Lasswell, 1948). A social communication process starts with a stimulus, which is an idea or a concept that is activated by the sender. The audience members may record the message after receiving the information. The effect of information communication means that the cognition, attitude, emotions, and behaviors of the audiences may change during information processing. Cognition reflects the conscious thinking processes of receiving information from the environment, synthesizing that information, and formulating actions based on the synthesis (Walsh, 2018). In addition, when audiences receive any information, they may experience a mental process of information processing (Shannon and Weaver, 1963). Based on the dual-process theories of information processing, information processing entails both heuristic and systematic processing (Chaiken et al., 1989; Eagly and Chaiken, 1993; Kievik and Gutteling, 2011; Wong et al., 2021). In the heuristic mode, individuals are likely to perceive information delivered by experts or endorsed by others and may make light of the detailed assessment of information to form a judgment, especially in events or situations of uncertainty. Systematic processing permits individuals to employ a full evaluation of the information. The ability of information processing is how individuals perceive, judge, evaluate, and think about information. During the COVID-19 pandemic, online information reporting is growing exponentially. The role of media exposure has also been associated with public anxiety levels during the COVID-19 pandemic (Liu et al., 2020). The public can make better-informed decisions when they have access to and understand further information (VanderWeele, 2020). Therefore, this study planned to analyze public reactions to the COVID-19 pandemic by investigating stimuli, public self-perceptions, abilities of information processing, and cognition from the data of Sina Weibo.

Social media plays a significant role in the propagation and health communication of messages, which may influence public mental processing and coping. Therefore, the aims of this study are (a) to capture the trajectory of psychological status during the COVID-19 pandemic by using Weibo posts; (b) to analyze public sentiments and public opinion regarding the COVID-19 pandemic and some important events with high public attention posts from social media; (c) to summarize the status of external stimuli, public self-perception, information processing, and cognition to the COVID-19 pandemic from a media perspective; and (d) to propose a crystallization framework, which can provide a reference for other counties and in the future to promote public mental health during the COVID-19 pandemic and other major, sudden infectious diseases.

## Materials and Methods

### Data Source and Procedure

#### The Process of the Collection of Newly Confirmed COVID-19 Cases

We collected the number of newly confirmed cases by week from December 30, 2019, to May 25, 2020, from the National Health Commission of the People's Republic of China (http://www.nhc.gov.cn/xcs/yqtb/list_gzbd_23.shtml). Due to a change in statistical methods on February 12, 2020, the number of newly diagnosed cases increased significantly on that day (i.e., 15,152 cases were diagnosed in a single day). To reduce the interference of abnormal nodes, the number of newly confirmed COVID-19 cases on February 12 was set as 3,552, which was the average of 2 days before and after based on the simple moving average smoothing method (Yang et al., 2019; Ediriweera et al., 2020; McGovern, 2020).

#### The Process of the Collection of Weibo Posts

The public sentiment data were derived from the Harbin Institute of Technology Joint Laboratory for social networks and a data mining research team called WeiboReach, which is one of the most advanced new media (including Weibo, WeChat, and other online media) news propagation analysis tool in China. This study used information from Sina Weibo, which is a social media platform in China. Sina Weibo is the most widely used social media platform for Internet users in China for sharing and discussing individual attitudes, opinions, events, and activities publicly. The data of Sina Weibo are emerging as a key online media and data source for researchers to understand social problems in a non-invasive way. Using keywords in Chinese, such as “(Wuhan and Severe Acute Respiratory Syndrome), (Wuhan and SARS), (Wuhan and pneumonia), (South China Seafood and pneumonia), (Wuhan and coronavirus), novel coronavirus, (Wuhan and lockdown), major public emergencies health events, (novel and pneumonia), (epidemic and resumption of work), (COVID-19 and epidemic), (2019 novel coronavirus and epidemic), (COVID-19 and pandemic), and (2019 novel coronavirus and pandemic),” this study applied a keyword maximization search method to obtain Weibo posts from December 30, 2019, to May 25, 2020 (i.e., the first wave of the COVID-19 pandemic in China). The WeiboReach database accumulated 304,757 Weibo posts, including original and forwarding posts, in response to the COVID-19 pandemic during the first wave of the COVID-19 pandemic in China. For this study, limited by the consideration of funds and the amount of data for analysis, 5–10% of Weibo tweets were randomly selected at equal intervals during the period of the first COVID-19 pandemic in China. We also ensured that an average of at least 100 Weibo posts per day was selected. Ultimately, a total of 20,114 Sina Weibo posts were selected by the equidistant random sampling method. The final sampling ratio was 6.6%. This study included Weibo posts published by the lay public and organizations such as media and government officials.

We conducted the social media sentiment analysis through natural language processing (NLP) in this study. To explore the emotions of Internet users toward COVID-19, we assessed sentiment inclination through big data screening (e.g., automatic clustering, emotion determination, and other machine algorithms) and manual determination.

#### The Preprocessing for the Acquired Initial Text Data

To ensure the accuracy of automatic sentiment analysis, we conducted data preprocessing, which contained data cleaning, and word separation. We used Python (Python Software Foundation) to perform data cleaning. We could effectively remove the error information of the data through noise removal. We removed all stop words, typos and garbled characters, and the special characters from the text. To calculate the emotions of Internet users, we excluded Weibo posts from organizations such as media, companies, and government officials. In terms of the textual content of Weibo posts, we removed posts that simply forwarded and shared the content of news websites and posts that were unrelated to the pandemic-related topics. In addition, some posts with the same text posted by the same user were removed during the data cleaning process. This study examined as many COVID-19 pandemic-related topics as possible. Then, we used the Python package Jieba for word separation. We divided complete sentences into a collection of meaningful symbols by word separation. After the preprocessing of data cleaning and word separation, this study converted the text into words, which can be computed into numeric vectors in the next procedure.

#### Using a Pretrained Model to Conduct Feature Extraction and the Classification of Public Emotional Postings

To conduct the public sentiment-type analysis, we used a pretrained model that combines the advantages of one-hot representation and word2vec to conduct feature extraction to convert the words into numeric vectors. The pretrained model solves the problem of the high subjectivity of the polarity scores of emotional words in the traditional sentiment dictionary method. Some studies have shown that the machine-learning-based methods are more effective at classification than the sentiment dictionary methods (Tan and Zhang, 2008; Tanana et al., 2021). Recently, more popular pretraining models have been the bidirectional encoder representations from transformers (BERT) model and its improved versions RoBERTa and RBT3 (Liu et al., 2019). The Joint Laboratory of HIT and iFLYTEK Research (HFL) released the RBT3 model in early 2020, which is a shortened version of the RoBERTa-wwm-ext model. It is only 37.3% of the volume of the first-generation model, but the actual effect is similar to the BERT model. The RBT3 model vectorizes words by calculating the position and meaning of words and sentences in the text and is further combined with the deep learning techniques to achieve sentiment analysis. Thus, this study used the RBT3 model to discriminate public sentiment during the COVID-19 pandemic.

To classify public emotional postings, an effective classifier was built by using a dataset with million-level Sina Weibo posts. It was modeled as a supervised learning process. A proportion of 70% of the dataset was selected as the training set, and 30% of the dataset was selected as the test set. The results of the training set were used to reprocess the whole dataset and to calculate the final sentiment orientation, and the results of the test set were used to calculate accuracy. The accuracy of the automatic sentiment analysis was 81%. Further details about the identification of public emotional posting by a supervised learning process have been described elsewhere (Tian et al., 2016; Wang et al., 2018; Yao et al., 2020, 2021). All sampled 20,114 Weibo posts were automatically classified with “optimistic orientation,” “pessimistic orientation,” or “neutral orientation” tags. Posts such as go, hope, bless, peace, praise, good news, preventable and controllable, joy, happy, and other emotional words were tagged as indicating an optimistic orientation, while posts such as the words in action, concealment, opaqueness, anger, horror, fear, frightening, and other words with obvious negative emotional valence were classified as pessimistic orientation.

To further improve the accuracy of the automatic sentiment analysis and guarantee the subsequent analysis of the data, this study further manually tagged those posts that were initially classified as “pessimistic orientation” and “neutral orientation” according to the actual experimental results and the high accuracy rates for optimistic texts. In this study, two experts with more than 5 years of work experience in the judgment of public opinion manually tagged the emotional orientations separately. A third expert then checked the consistency of the results and determined the final results.

#### Calculating the Proportion of Sentiment Orientation Tags

To understand the trajectory of psychological status during the first wave of the COVID-19 pandemic in China, the proportion of optimistic orientation tags and pessimistic orientation tags in the total sample data by week was calculated. The final sentiment proportion ranged from −100% to 100%. Specifically, 0–100% was the percentage of optimistic tendency, while −100% to 0 was the percentage of pessimistic tendency. To reduce the interference of abnormal nodes (e.g., February 12, 2020), we calculated the proportion of the emotional orientation of Internet users using the average of 2 days before and after based on the simple moving average smoothing method (Li and Wei, 2016; Yang et al., 2019; Ediriweera et al., 2020).

#### Selecting Important Events Regarding the COVID-19 Pandemic to Analyze Public Emotions to Government Measures and Major Events

To analyze public emotions to government measures and major events with high online influence pertaining to the COVID-19 pandemic, we selected events according to the event impact index (EII) and the policies of government decision-making events regarding the COVID-19 pandemic prevention and control during December 30, 2019, to May 25, 2020 (i.e., the first wave of the COVID-19 pandemic in China). The EII was based on the data of both social media and network media for the whole network and was used to describe the transmission effect of a single event on the Internet. The EII was calculated by taking into account the position of the keywords (i.e., Weibo headline or Weibo posts content), the weight of communication channels (i.e., Weibo, WeChat, and other online media), and the weight of event attributes. The communication effects of events on social media and online media were summarized, and then, the event influence was normalized to obtain the EII, which ranged from 0 to 100. Since the EII of an event exceeds 65, it is generally necessary to perform emergency management for this event. We included these events regarding COVID-19 during the first wave of the COVID-19 pandemic in China with the EII more than 65 in this study. In addition, to improve the understanding of public policy literacy, we still included an anti-epidemic strategy (i.e., The Central Leading Group for COVID-19 Response held a meeting to deploy an initiative for guarding against imported cases and a rebound in indigenous cases), even though the EII of it did not exceed 65. Finally, 15 events were included in this study, with 7 government decision-making events regarding the COVID-19 pandemic prevention and control and 8 major events regarding COVID-19. If the number of Weibo posts in each event was <1,000, all the posts were included; if not, the posts of each event were randomly sampled at a rate of 10–20% due to possible loss.

#### Selecting the Top Two OP and PO Events to Analyze Public Opinion

**Figure 1:**
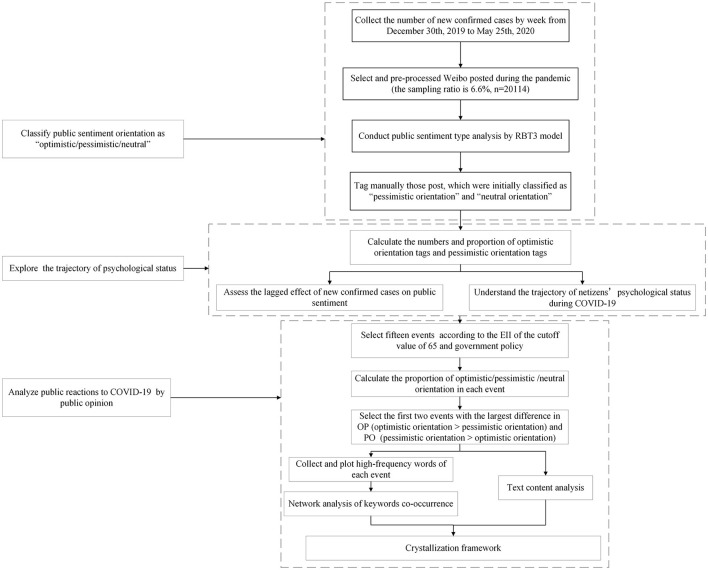
Flow diagram of this study.

Finally, the proportions of optimistic orientation, pessimistic orientation, and neutral diagram of this study orientation and the differences among them were calculated within each event. We focused on the expression of publicly optimistic and pessimistic emotions. Thus, the events were further divided into the proportion of optimistic orientation > the proportion of pessimistic orientation (OP) events and the proportion of pessimistic orientation > the proportion of optimistic orientation (PO) events. The top two OP events and two PO events with the largest difference in the proportion of optimistic orientation and pessimistic orientation were selected for textual content analysis. The high-frequency words in Weibo were collected and plotted. To further analyze public reactions in the two PO and two OP events, we supplemented them with the manual content analysis. To further analyze public reactions in the two PO and two OP events, we supplemented them with manual content analysis. We conducted a network analysis of keyword co-occurrence in each of these four events to further explore the possible links and occurrence among these high-frequency words. The flow diagram is shown in Figure 1.

### Construction of the Coding Scheme

We developed the coding scheme based on the top two PO events with the largest difference in the proportion of pessimistic orientation and optimistic orientation (No. 6: Shuanghuanglian Oral Liquid could inhibit COVID-19; No. 8: Dr. Wenliang Li, the pandemic whistleblower, died of COVID-19) and the top two OP events with the largest difference in the proportion of optimistic orientation and pessimistic orientation (No. 11: The Academy of Military Medical Sciences (AMMS) of the People's Liberation Army (PLA) Academy of Military Science successfully developed a recombinant vaccine for COVID-19; No. 13: The national medical team supporting Hubei began to evacuate in March 17). The textual content analysis was performed for all the Weibo posts related to posts of the selected four events based on the anxiety theory suggested by Edith Jacobson (Tuttman, 1985). The Weibo posts were first classified as the stimulus, self, ability, and cognition, which were defined as the first-level categories of coding. These Weibo posts related to news reporting, expert responses, event or information propagation, social phenomena, and herd behaviors were coded as stimuli. Weibo posts conveying the personal emotions of the publisher were coded as Self. If the Weibo posts reflected a mental process of information processing by the publisher, these posts were classified as ability. The information processing ability included information dissemination, judgment, evaluation, and thinking. Weibo posts revealed that the publishers had formulated individual attitudes, attribution, and actions after the conscious thinking processes were coded as cognition. The second-level categories were subdivided under the first-level categories. We further provided an explanation for the content of secondary categories according to each Weibo post for the two OP events and the two PO events. The detailed coding table is shown in Table 1. The coding manual was used to create the coding table of public reactions.

**Table 1:** Coding table of Weibo content.

**First-level category**	**Second-level category**	**Explanation**	**Coding**
Stimulus			
	News	Direct or related reports of the incident	Aa
	Phenomenon stimulus	Reports of the public behavior triggered by the incident	Ab
	Event propagation	The indirect spread of the event	Ac
Self			
	Positive perception	A positive feeling toward the event	Ba
	Negative perception	A negative feeling toward the event	Bb
	Neutral perception	A neutral reaction to the event.	Bc
Ability			
	Information dissemination	The ability to convey a message, idea, attitude, or emotion to another person or group	Ca
	Judgment	The skill and ability to analyze, distinguish and judge things through thinking	Cb
	Evaluation	The ability to correctly evaluate information and its sources and to make effective use of it	Cc
	Thinking	The ability to take a broad view of the problem, think beyond boundaries, and to integrate, screen, and supplement information	Cd
Cognition			
	Positive opinion	A positive view of the incident	Da
	Negative opinion	A negative view of the incident	Db
	Neutral opinion	A neutral view of the incident	Dc

### Coding Process

This study initially conducted the textual content analysis by four researchers divided into two groups. When each group randomly analyzed an event, the two members of this group first coded it separately. A third researcher then judged the consistency of the coding results. Intercoder reliability was measured using Cohen's kappa. The kappa coefficient was 0.86 in this study, which indicated that the consistency of the results of manual coding was very good (Altman, 1991). Then, all of the researchers discussed and analyzed any inconsistent results until they attained agreement. Each Weibo post could be classified into different first-level categories but only one second-level category.

### Data Analysis

This study employed the *t*-test method to compare the differences between publicly optimistic and pessimistic sentiment during the three periods of COVID-19: the information uncertainty period, the outbreak attention period, and the regular epidemic prevention and control period. We further used lag-1 to lag-8 weeks to examine the lagged effect on the number of posts with pessimistic/optimistic orientation and the difference between them in the weekly number of COVID-19 cases by the Pearson's correlation coefficient. We used a proportional *Z*-test to analyze the difference in the proportions of Weibo posts classified as each second-level category for events No. 6/No. 11/No. 13 in comparison with event No. 8 among self-perception, ability, and cognition. We used Microsoft Excel (Microsoft Corporation, One Microsoft Way Redmond, WA 98052-6399, USA), SPSS software version 21.0 (SPSS Inc., Chicago, Illinois, USA), SAS version 9.4 (Copyright © SAS Institute Inc., SAS Campus Drive, Cary, North Carolina 27513, USA. All rights reserved.) for the statistical analyses, and NVivo version 12.0 (QSR International, Burlington, MA) for the manual content analysis. The network analysis of keyword co-occurrence was conducted with the R package igraph (Free Software Foundation Inc.) (Csardi and Nepusz, 2006).

## Results

### Trajectory of Psychological Status During the Three Periods of the COVID-19 Pandemic

**Figure 2:**
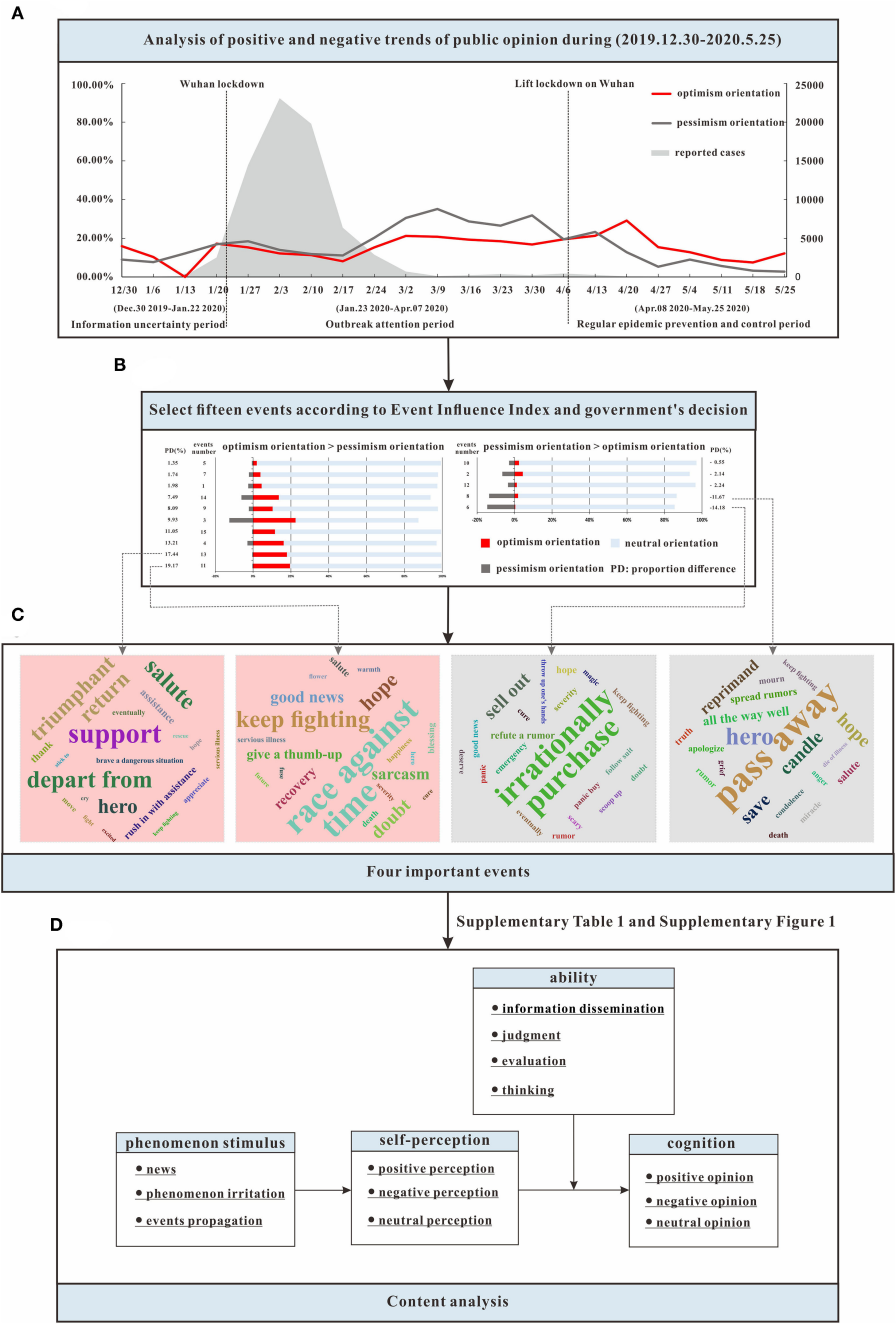
Study design and results. **(A)** Showed the proportion of public optimistic orientation (the red curve), pessimistic orientation (the gray curve) and the newly confirmed COVID-19 cases (the gray shade) by week from December 30th, 2019, to May 25th, 2020; **(B)** indicated public emotional reactions to 15 important events; **(C)** revealed the results of word clouds; and **(D)** exhibited our crystallization framework.

The two curves in Figure 2A showed the proportion of public optimistic orientation (i.e., the red curve) and pessimistic orientation (i.e., the gray curve) by week from December 30, 2019, to May 25, 2020, which were classified from postings. The gray shade in Figure 2A indicated the newly confirmed COVID-19 cases by week from December 30, 2019, to May 25, 2020. We used the *t*-test method to analyze public emotional reactions during the three periods of COVID-19. During the information uncertainty period, the proportion of posts with an optimistic/pessimistic orientation initially indicated a downward trend, followed by an upward trend. There was an intersection in the proportion of posts with an optimistic orientation and those with a pessimistic orientation on January 6 (i.e., the first appearance of the topic “Wuhan pneumonia of unknown cause” in Weibo). After that date, the optimistic orientation dropped lower than the pessimistic orientation, but the difference was nonsignificant (*t* = 0.056, *P* > 0.05). During the outbreak attention period, the pessimistic orientation was higher than the optimistic orientation, but the difference was also non-significant (*t* = −0.948, *P* > 0.05). Before February 17 (i.e., the initiation of the rapid series of responses of the Chinese government), both the optimistic and pessimistic orientations showed a constant downward trend. After that date, both began to trend upward. During the regular epidemic prevention and control period (after the lockdown in Wuhan was lifted), the pessimistic orientation rose briefly at first and then fell, and the optimistic orientation was significantly higher than the pessimistic orientation (*t* = 3.571, *P* < 0.05).

### The Time-Lag Effect of Newly Confirmed COVID-19 Cases on Public Optimistic/Pessimistic Orientation

**Figure 3:**
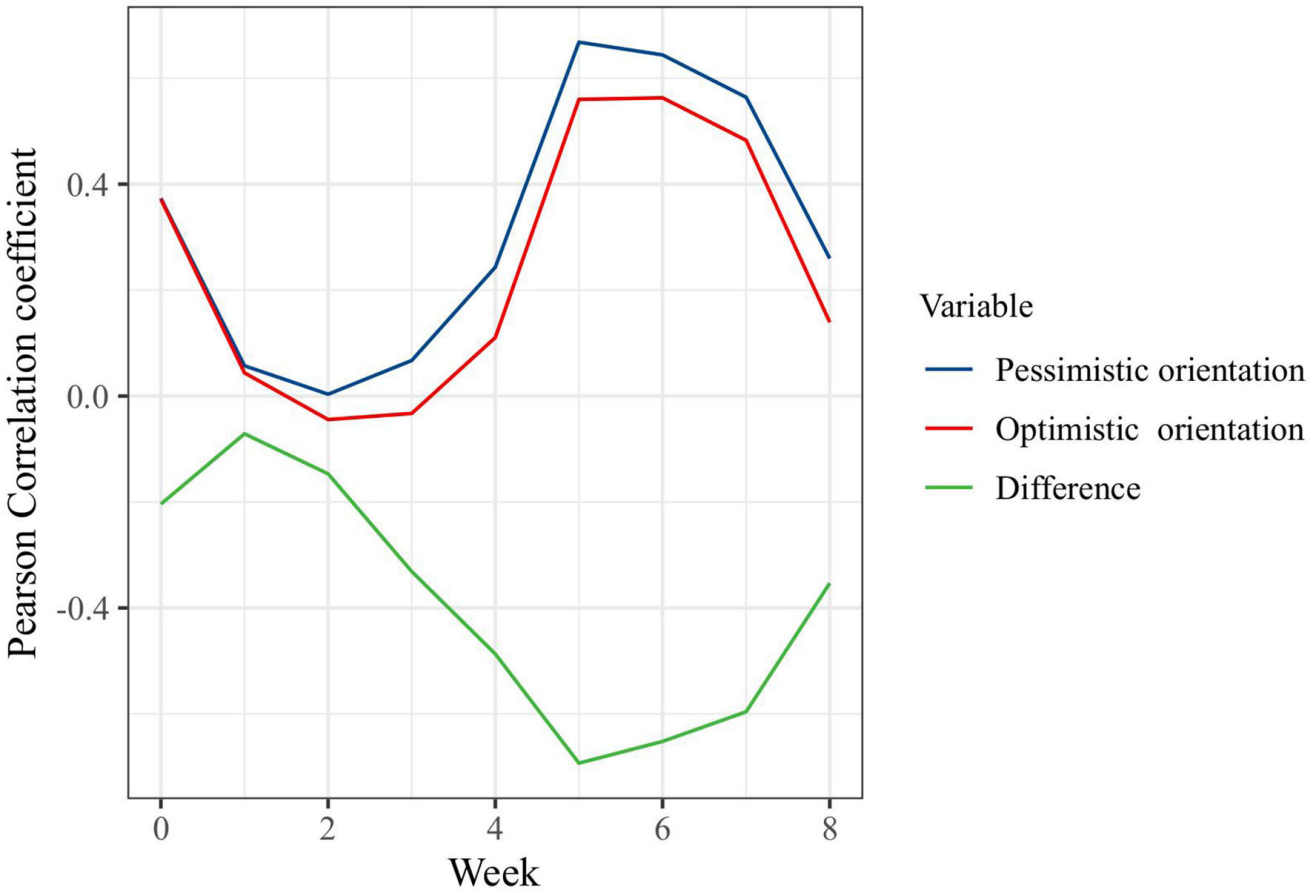
Pearson's correlation coefficient between the newly confirmed COVID-19 cases and public pessimistic/optimistic orientation. “Pessimistic orientation” indicates the number of posts with pessimistic orientation, while “Optimistic orientation” indicates the number of posts with optimistic orientation. “Difference” indicates the difference between the numbers of posts with optimistic orientation and pessimistic orientation.

Pearson's correlation coefficient was used to analyze the lagged effect of the newly confirmed COVID-19 cases on the public pessimistic/optimistic orientation. The number of newly confirmed COVID-19 cases had a lagged effect on the public optimistic sentiment, pessimistic sentiment, and difference between the two. The correlation coefficient between the newly confirmed COVID-19 cases and public sentiment decreased, increased, and then decreased over time, forming an S-shaped curve. That is, the number of newly confirmed COVID-19 cases had a significant positive correlation with optimistic sentiment at a delay of 5–6 weeks and a significant positive correlation with pessimistic sentiment at a delay of 5–7 weeks. There was a significant negative correlation with the difference in posts containing optimistic and pessimistic sentiment at a delay of 4–7 weeks (Figure 3). As the number of newly confirmed cases decreased, the optimistic orientation grew to be higher than the pessimistic orientation.

### Sentiment Analysis of Important Events

During the three periods under study, we chose 15 important events (Table 2). Among the events, there were 10 OP events (2/3) and 5 PO events (1/3). The events were sorted according to the difference in the proportion of posts with optimistic orientation and posts with pessimistic orientation (Figure 2B). For most of these events, the proportion of posts with neutral orientation was over 80%. Notably, for event No. 3 (i.e., Wuhan lockdown), the proportion of posts with neutral orientation was the lowest (64.97%) and the proportion with optimistic orientation was the highest (22.48%). For event No. 6 (i.e., Shuanghuanglian Oral Liquid could inhibit COVID-19), the proportion with pessimistic orientation was the highest (14.59%). For event No. 13 (i.e., The national medical team began to evacuate), the difference was the highest (17.44%).

**Table 2:** The 15 important events.

**Events**	**Name**	**Optimistic** **orientation** ***n* (%)**	**Pessimistic** **orientation** ***n* (%)**	**Neutral** **orientation** ***n* (%)**	**Total**	**Event influence index**
No. 1	The Wuhan Municipal Health Commission was notified of 1 death case, which the experts interpreted as viral pneumonia of unknown cause	39 (4.55)	22 (2.56)	797 (92.89)	858	66.4
No. 2	The public began to pay attention to the scientific recommendations for preventing the pandemic, such as wearing a mask and washing hands regularly	64 (4.41)	95 (6.55)	1,291 (89.03)	1,450	79.6
No. 3	Wuhan announced its lockdown	326 (22.48)	182 (12.55)	942 (64.97)	1,450	84.5
No. 4	Wuhan built new makeshift hospitals according to the Xiaotangshan model	234 (16.18)	43 (2.97)	1,169 (80.84)	1,446	89.7
No. 5	The General Office of the State Council issued a notice to extend the Spring Festival holiday in 2020	31 (1.99)	10 (0.64)	1,516 (97.37)	1,557	73.9
No. 6	Shuanghuanglian Oral Liquid could inhibit COVID-19	6 (0.41)	211 (14.59)	1,229 (84.99)	1,446	79.1
No. 7	Public health experts predicted that the peak would probably occur on February 21st	77 (3.93)	43 (2.20)	1,837 (93.87)	1,957	74.1
No. 8	Dr. Wenliang Li, the pandemic whistle-blower, died of COVID-19	28 (1.90)	200 (13.57)	1,246 (84.53)	1,474	84.8
No. 9	The joint prevention and control mechanism of the State Council established the paired assistance method: 19 provinces mobilized to alleviate pressure on cities in Hubei Province other than Wuhan	147 (10.34)	32 (2.25)	1,243 (87.41)	1,422	68.1
No. 10	A new paper by Dr. Nanshan Zhong's research team reported that the incubation period of COVID-19 could be as long as 24 days	35 (2.39)	43 (2.94)	1,386 (94.67)	1,464	73.9
No. 11	The Academy of Military Medical Sciences (AMMS) of the People's Liberation Army (PLA) Academy of Military Science successfully developed a recombinant vaccine for COVID-19	410 (19.55)	8 (0.38)	1,679 (80.07)	2,097	69.5
No. 12	The WHO declares that COVID-19 has pandemic characteristics	21 (1.24)	59 (3.48)	1,614 (95.28)	1,694	75.8
No. 13	The national medical team supporting Hubei began to evacuate on March 17th	256 (17.93)	7 (0.49)	1,165 (81.58)	1,428	90.6
No. 14	The Central Leading Group for COVID-19 Response held a meeting to deploy an initiative for guarding against imported cases and a rebound in indigenous cases	62 (13.66)	28 (6.17)	364 (80.18)	454	38.5
No. 15	The lockdown on Wuhan was lifted	168 (11.60)	8 (0.55)	1,272 (87.85)	1,448	89.6

### Analyzing Online Public Reactions Through Textual Content Analysis and Network Analysis for Specific Events

The top two OP and PO events were selected for further analysis. We identified the optimistic orientation and pessimistic orientation words that appeared most frequently for each event and visualized them using word clouds (Figure 2C). Among the PO events, the word that occurred most frequently for event No. 6 was “irrationally purchase,” which occurred 548 times; for event No. 8, the most frequently occurring word was “pass away,” which occurred 1,546 times. Among the OP events, the most frequently occurring word for event No. 11 was “race against time,” which occurred 41 times; the most frequently used word for event No. 13 was “support,” which occurred 1,947 times.

The analysis of the high-frequency word graphs for these four specific events showed that the same high-frequency words occurred for different events; however, the semantics might differ depending on the situation. For example, “hope” occurred in both OP event No. 11 and PO event No. 6; in the former context, it was related to breakthroughs in the pandemic, while in the latter context, it was related to the hope of the public, which Dr. Li would be rescued. The high-frequency word “hero,” which occurred in OP event No. 13 and PO event No. 8, represented the respect and support of the public for medical staff. This emphasis was inseparable from the declarations and efforts of the Chinese government to promote the image of doctors, using words such as sincere, kind, and admirable, which prompted the diffusion of “positive energy” throughout the public during the pandemic to encourage pandemic prevention and control efforts.

The stimulus, public self-perception, ability, and cognition situations are shown in Supplementary Table 1. A proportional *Z*-test was used to analyze the difference of events No. 6/No. 11/No. 13 in comparison with event No. 8 among self-perception, ability, and cognition. There was a difference in the proportion of Weibo posts classified as different second categories among events No. 8, No. 6, and No. 11 for self-perception and cognition (*P* < 0.05). The proportion of Weibo posts classified as primary ability and advanced ability was different among events No. 6, No. 13, and event No. 8. The proportion of advanced ability was higher in event No. 8 (PO) than in event No. 13 (OP). The public tended to develop more negative perceptions of PO events (No. 8) than OP (No. 11 and No. 13) events. However, the proportion of Weibo posts with positive opinions might not be low if the public had more advanced judgment and thinking abilities.

To supplement the explanation of the phenomenon, we conducted a network analysis of key word co-occurrence in each of the four events. We examined which keywords frequently co-occur in opinion context with one another. The results of four networks of the high-frequency words are shown in Supplementary Figure 1. In the map, the lines show the relationships between keywords, and line thickness indicates the frequency of keyword occurrence. For example, in the PO event No. 6 (i.e., Shuanghuanglian Oral Liquid could inhibit COVID-19), we found that the keyword “irrationally purchase” coupled with public negative self-perception (words such as severity, magic, crazy, and panic) could also be linked with public positive self-perception (words such as keep fighting, deserve, defeat, well, and cure). The keyword “irrationally purchase” linked to the keyword “refute a rumor” *via* “rumor” and to “doubt,” “profit from national disaster,” and “insist” through the keyword “sell out.” The keyword “irrationally purchase” was linked to the keyword “effort” *via* “panic.” In the OP event No. 13 (i.e., The national medical team supporting Hubei began to evacuate on March 17), the keyword “triumphant return” coupled with public emotions (words such as salute, thank, move, excited). The keyword “triumphant return” was linked to the keyword “hope” *via* the keyword “move” and was related to “support” *via* “hero.” Thus, the external stimulation of irrational purchases and other information may be linked with public self-perception. After the different information processing tasks of dissemination and judgment, the cognition of people toward the external event stimulation may also differ (i.e., doubts about the news, refute a rumor, doubt the effect, insist, and hope for the epidemic).

The emotional ABC theory includes three elements, namely, activating events (A), the belief system (B), and the behavioral consequences (C) (Ellis, 1984). Our emotions and behaviors (C: Consequences) are not directly determined by events (A: Activating Events) but rather by the rational or irrational belief concerning these events (David et al., 2009). Referring to the emotional ABC theory, and the communication model suggested by Lasswell, we proposed a crystallization framework (of the public psychological process and reactions) based on the results of the textual content analysis and network analysis. We proposed a hypothesis that people develop different self-perceptions when they receive external stimuli. Then, depending on their abilities at the primary level (e.g., information dissemination) or advanced level (e.g., judgment, evaluation, and thinking) to decode the messages, their final cognitions may differ (Figure 2D).

## Discussion

This study used Sina Weibo posts to analyze the public psychological status regarding COVID-19 and some important events during the COVID-19 pandemic. We understood the topics that the public most frequently discussed by identifying their high-frequency words during the top two OP and PO events. This study also explored public psychological processes and reactions based on the network analysis of co-occurring keywords and manual textual analysis. We then formatted a crystallization framework (of the public psychological process and reactions).

The public psychological status differed among different periods of the COVID-19 pandemic; this result was consistent with other studies (Li Q. et al., 2020; Li Y. et al., 2020; Zhao et al., 2020; Yin et al., 2021). This study also illustrated the decline of pessimistic sentiment during the regular epidemic prevention and control period, which has also been revealed by other scholars (Jia and Liu, 2021). Positive emotions were higher than negative emotions in that period. During this period, public attention shifted to other aspects of the pandemic, and public opinions primarily expressed praise for the pandemic control achievements and tributes to healthcare workers of China (Li Q. et al., 2020). In addition, the results of this study showed that the newly confirmed cases of COVID-19 were related to changes in public psychological status and had an almost 1-month lag effect on public sentiment. Therefore, it is critical to improve public negative emotion monitoring and early warning mechanisms and to innovate emergency response methods for Internet public opinion.

Among the 15 events, two-thirds were trended toward an optimistic orientation. Amid the mass panic in response to the first reports of the pandemic, China was able to efficiently respond to the negative public perception and positively utilize effective information from public opinion monitoring to support pandemic control efforts. The Chinese government pays attention to and is responsive to the emotions of the citizens through social media (Dai et al., 2021). This result indicated that the risk communication strategy and public opinion guidance measures of China played a positive role in regulating the emotions of the public and that the strategies such as quarantines and city lockdowns have not had unduly negative effects on the public. This success was the result of the joint efforts of three parties, namely, the Chinese government, the news media, and the public. For example, during the lockdown of Wuhan, a large quantity of medical and living supplies were brought into the city, which provided for the safety and basic life needs of the population, which in turn helped the population appreciate the support they received from the larger community and encouraged them to feel that their situation was changing for the better (Vijayaraghavan and Singhal, 2020); they no longer wanted to escape because they no longer felt panicked about living in the city. The news media also published the latest news on Wuhan every day, and the increased use of the word “social” in Wuhan after the lockdown suggests a focus on social concerns and social support (Su et al., 2020). Another study also found that health agencies that fully utilize social media engagement strategies may be important to craft more interactive and engaging communications during a crisis (Gurman and Ellenberger, 2015). Additionally, the timely and fair dissemination of information enhanced the health literacy and policy literacy of people, helping them realize the necessity of national initiatives for promoting healthy behaviors.

According to the textual content analysis, we found that the public experienced high anxiety and stress due to the uncontrollable nature of the pandemic, the spread of rumors and misinformation, viral infections or deaths of medical staff, and panic buying. A study also found that negative public emotions, such as fear, anger, and sadness, were associated with rumors (Dong et al., 2020). The rapid dissemination of rumors and misinformation in new media can easily lead to the spread of public anxiety and can further increase the media and information literacy gap, resulting in greater differences in perception among groups within communities (Bates et al., 2020; Guo et al., 2020). The public needs to reduce their exposure to harmful messages about COVID-19 from news and websites and thereby eliminate negative external stimuli. Responsible social media use is important under any circumstances, especially during times of crisis (Hauer and Sood, 2020). In addition, health agencies should provide consistent updates about social media to build trust with the public and should release information about what is known and not known.

The ability to acquire information from authoritative institutions, discriminate rumors from facts, and critically assess information may be important for the promotion of positive cognition, although negative perceptions may arise from the spread of rumors and the unwise wording and behaviors of other people. Due to the external factors such as viral infection and the deaths of medical personnel, the public experienced negative emotions; however, after rational judgment, evaluation, and thinking, they showed increased gratitude and respect for doctors and trust in the government, and thus, they were not completely overwhelmed by these negative emotions. The dissemination of information about OP events increased the positive emotional perception of public, prompting them to be proud and happy about the scientific and technological progress of the country and the unity and cooperation of various communities; the resulting increase in positive emotions strengthened the belief of victory of the public in the fight against the pandemic. As a result, the public developed a positive perception and was more willing to adhere to national pandemic prevention and control measures.

At present, the COVID-19 pandemic has not been fully controlled globally, and some countries are facing a gradual worsening. In this situation, it is necessary to pay close attention to the mental health and emotional changes in patients with COVID-19 and other members of society while engaging in risk communication. The digitally networked global public likely affected discourse, sentiment, and response online (Roberts et al., 2017). To improve the capability of the public to judge truth and e-health literacy and to increase the breadth and depth of information communication, official authorities/opinion leaders/experts need to deliver high-quality health information and policy implications. Professionals at healthcare institutions should utilize effective information on public opinion to reduce stress and improve risk perception for the public, thereby enhancing the emotional response capabilities of the public (Chan et al., 2018; Malecki et al., 2020). At the same time, we should also encourage all citizens to participate and work together toward hope and optimism so that the pandemic can be contained (World Health Organization, 2020a). Only in this way people can benefit worldwide from the introduction of health promotion policies and plans. As the WHO director general stated, the strategies China has implemented are good not only for China but also for the rest of the world (World Health Organization, 2020b).

### Limitations and Strengths

There were several limitations to this study. First, we analyzed Sina Weibo posts from the first phase of the pandemic in China (from December 2019 to May 2020); however, there are other social platforms that the public may also have engaged with. Moreover, not all members of the public use Weibo. Second, our results may not reflect the present and future public opinion. Finally, we used a computation method to analyze online content and supplemented it with qualitative analysis to examine public reactions to the COVID-19 pandemic in China. We proposed a hypothesis based on the results of the textual content analysis and network analysis. However, the results of this study cannot indicate causal effects, although we analyzed the possible links between high-frequency words in each of two OP and two PO events through the network analysis of keyword co-occurrence. Further quantitative survey analysis is needed to explore the relationships between external stimuli, self-perception, information processing ability, and cognition and to research the influencing mechanism. This study explored public psychological status and reactions regarding the COVID-19 pandemic using qualitative and quantitative analysis, which should be further validated in the future by public opinion on other similar public health emergencies.

Despite there were those limitations, this study had some practical contributions to health management. This study understood the trajectory of psychological status during the first 6 months of the COVID-19 pandemic in China and analyzed public opinions on important events regarding the COVID-19 pandemic. It can provide evidence-based public health communication approaches for health agencies in China and other countries. We recommend that the health agencies pay attention to promote public risk perception, cognitions, and coping abilities, thereby improving the mental health of the public during the COVID-19 pandemic.

In addition to practical implications, this study also contributed to existing literature. This study contributed to the current understanding of the simultaneous and lagged effect of the newly confirmed COVID-19 cases on public emotional reactions. In addition, this study proposed a crystallization framework, which extended the emotional ABC theory and the communication model suggested by Lasswell in the context of the emotions of Internet users to the public health emergency, such as the COVID-19 pandemic. The audience members may record the message after receiving the information. When people encounter external stimuli, they may develop different emotions. Then, the final effect of the message such as the cognition, attitude, and behaviors of the audiences may change depending on their information processing ability to decode the messages.

## Conclusion

This study analyzed the trajectory of psychological status and public reactions during the COVID-19 pandemic by using the big data derived from the Sina Weibo posts. The public psychological status differed among different periods of the COVID-19 pandemic. The newly confirmed cases had an almost 1-month lagged effect on public psychological status. The public may have negative reactions to negative external stimuli. However, public cognition may be rational when these negative external stimuli are processed and evaluated. This study complements the existing findings of public emotions and reactions to the COVID-19 pandemic using the national dataset from Sina Weibo in China. This study also provides some implications for public health policymakers in understanding the emotional and cognitive reactions of the public to emergent public crises. Professionals at healthcare institutions should focus on reducing public pessimistic sentiment in the early period and improving the risk perception of the public based on the public opinion and the coping abilities of people, thereby benefiting by preventing the next possible major public event.

## Data Availability Statement

The datasets used and/or analyzed during the current study are available from the corresponding author on reasonable request.

## Ethics Statement

Ethical review and approval was not required for the study on human participants in accordance with the local legislation and institutional requirements. Written informed consent from the participants' legal guardian/next of kin was not required to participate in this study in accordance with the national legislation and the institutional requirements.

## Author Contributions

Y-CC had full access to all of the data in the study and took responsibility for the integrity of the data and the accuracy of the data analysis. Y-CC, TC, C-YL, MC, SL, and XC conceived of the study, participated in its design and coordination, and drafted the manuscript. QC and HW performed the data acquisition and sampling. AL helped to draft the parts of the manuscript. MC, SL, XC, AL, JR, XZ, and FX performed the data analysis. All authors approved the final version, took responsibility for its content, and read and approved the final manuscript.

## Funding

This study was supported by the Bill and Melinda Gates Foundation (Grant No. INV-005834 to TC) and the Scientific Research Grant of Fujian Province of China (No. Z0230104). The funder was not involved in the study design, collection, analysis, interpretation of data, the writing of this article or the decision to submit it for publication.

## Conflict of Interest

QC is employed by Nanjing Terlton Information Technology Co. Ltd., and HW is employed by Beijing Hongbo Zhiwei Technology Co. Ltd. The remaining authors declare that the research was conducted in the absence of any commercial or financial relationships that could be construed as a potential conflict of interest.

## Publisher's Note

All claims expressed in this article are solely those of the authors and do not necessarily represent those of their affiliated organizations, or those of the publisher, the editors and the reviewers. Any product that may be evaluated in this article, or claim that may be made by its manufacturer, is not guaranteed or endorsed by the publisher.

## Supplementary Material

Supplementary Table 1 The results of textual analysis.

Supplementary Figure 1 The results of network analysis of keyword co-occurrence.
