# Supplementary material for: Capturing the Trajectory of Psychological Status and Analyzing Online Public Reactions During the Coronavirus Disease 2019 Pandemic Through Weibo Posts in China
Source: Front Psychol. 2021 Sep 29;12:744691. doi: 10.3389/fpsyg.2021.744691 (PMC8511417; doi:10.3389/fpsyg.2021.744691)
Supplement: Supplementary Table 1 — The results of textual analysis. [file Table_1.pdf]

Supplementary Table 1 | The results of textual analysis.

Category	Important events			
	**No. 6 (PO) Shuanghuanglian Oral Liquid n (%)**	**No. 8 (PO) Dr. Wenliang Li died n (%)**	**No. 11 (OP) A recombinant vaccine for COVID-19 n (%)**	**No. 13 (OP) The medical team began to evacuate n (%)**
Stimulus	556	317	570	1142
News	322 (57.91)	267 (84.23)	570 (100)	1142 (100)
Phenomenon irritation	234 (42.09)	2 (0.63)	0 (0)	0 (0)
Event propagation	0 (0)	48 (15.14)	0 (0)	0 (0)
Self- perception	552 ***	734	279 ***	192 (NA)
**Positive perception**	64 (11.55)	145 (19.75)	**274 (98.21)**	**187 (97.40)**
**Negative perception**	**412 (74.37)**	**569 (77.52)**	3 (1.08)	5 (2.60)
Neutral perception	78 (14.08)	20 (2.72)	2 (0.72)	0 (0)
Ability	442 ***	38	17	30 *
Primary ability				
Information dissemination	119 (26.92)	2 (5.26)	2 (11.76)	4 (13.33)
Advanced ability	**323 (73.08)**	**36 (94.74)**	**15 (88.23)**	**26 (86.67)**
Judgment	323 (73.08)	12 (31.58)	13 (76.47)	8 (26.67)
Evaluation	0 (0)	0 (0)	0 (0)	18 (60.00)
Thinking	0(0)	24 (63.16)	2 (11.76)	0 (0)
Cognition	413 ***	114	22 *	20 (NA)
**Positive opinion**	**338 (81.84)**	**67 (58.77)**	**13 (59.09)**	**19 (95.00)**
Negative opinion	45 (10.90)	40 (35.09)	5 (22.73)	1 (5.00)
Neutral opinion	30 (7.26)	7 (6.14)	4 (18.18)	0 (0)

Note: We used proportional Z-test to analyze the difference of events No. 6/No. 11/No. 13 in comparison with event No. 8 among self-perception, ability, and cognition. *p<0.05; ***p<0.001; NA: Not Available (Since some cells were equal to 0).
