# Supplementary figures and images for: Capturing the Trajectory of Psychological Status and Analyzing Online Public Reactions During the Coronavirus Disease 2019 Pandemic Through Weibo Posts in China
Source: Front Psychol. 2021 Sep 29;12:744691. doi: 10.3389/fpsyg.2021.744691 (PMC8511417; doi:10.3389/fpsyg.2021.744691)

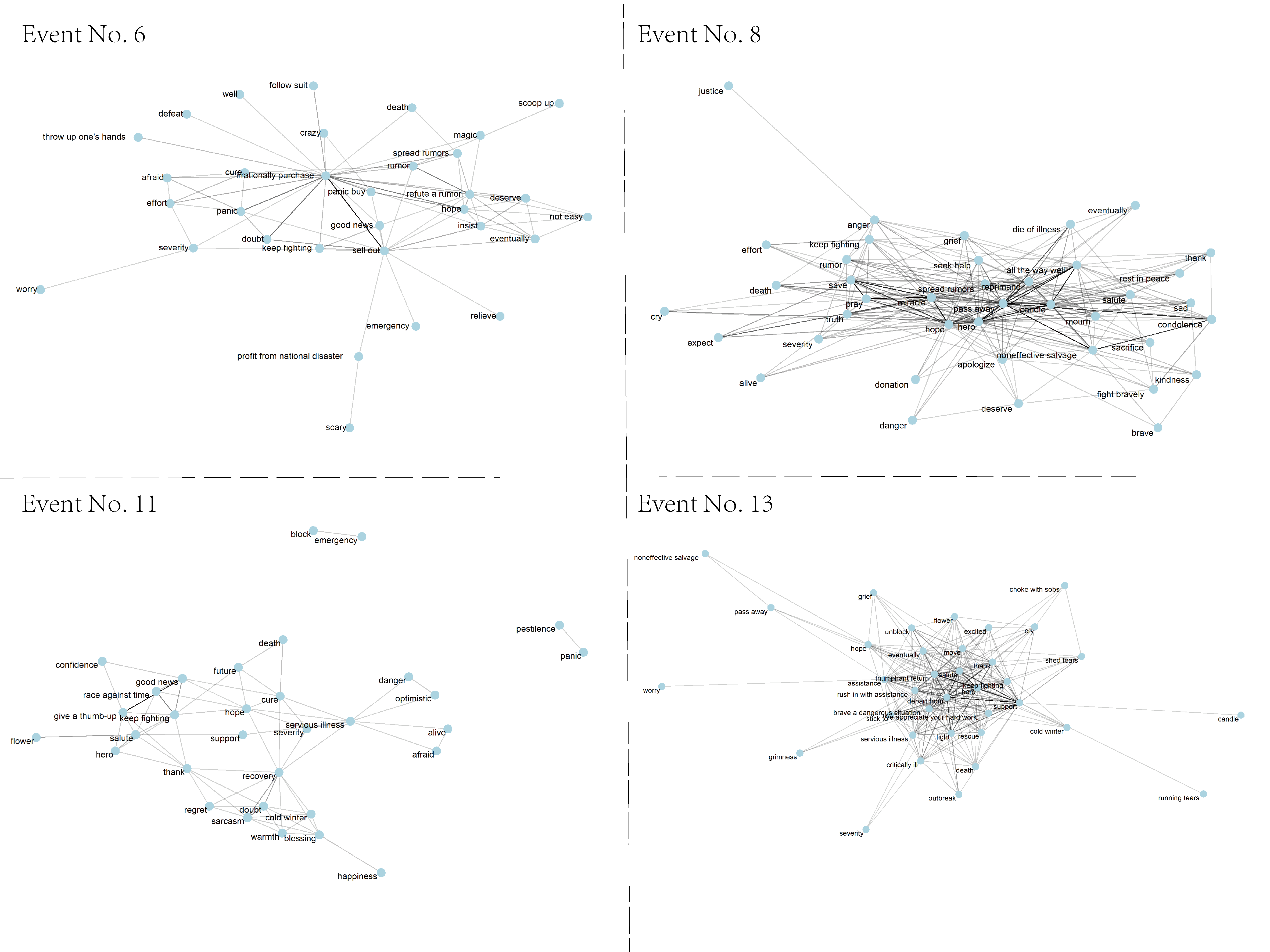

Supplement: Supplementary Figure 1 — The results of network analysis of keyword co-occurrence. [file Image_1.TIF]
